# Dinutuximab Beta Maintenance Therapy in Patients with High-Risk Neuroblastoma in First-Line and Refractory/Relapsed Settings—Real-World Data

**DOI:** 10.3390/jcm12165252

**Published:** 2023-08-11

**Authors:** Aleksandra Wieczorek, Urszula Żebrowska, Marek Ussowicz, Agnieszka Sokół, Marzena Stypińska, Bożenna Dembowska-Bagińska, Katarzyna Pawińska-Wąsikowska, Walentyna Balwierz

**Affiliations:** 1 Department of Pediatric Oncology and Hematology, Jagiellonian University Medical College, 30-663 Krakow, Poland; 2 Department of Pediatric Oncology and Hematology, University Children’s Hospital of Krakow, 30-663 Krakow, Poland; 3 Department of Pediatric Bone Marrow Transplantation, Oncology and Hematology, Wroclaw Medical University, 50-367 Wroclaw, Poland; 4 Department of Oncology, Children Memorial Health Institute, 04-730 Warsaw, Poland

**Keywords:** dinutuximab beta, maintenance therapy, high-risk neuroblastoma, relapsed, refractory, real-world data

## Abstract

Dinutuximab beta is approved for the maintenance treatment of patients with high-risk neuroblastoma (HR-NB), including patients with relapsed/refractory (R/R) disease. However, the data on its use in real-world clinical practice is limited. We retrospectively reviewed the clinical records of 54 patients with HR-NB who received maintenance therapy with dinutuximab beta in first-line (37 patients) or R/R settings (17 patients) at three centers in Poland. Of the 37 patients who received first-line treatment, twenty-eight had a complete response, two had a partial response, three had progressive disease, and four relapsed at the end of treatment. The median overall survival (OS) was 24.37 months, and the three-year progression-free survival (PFS) and OS were 0.63 and 0.80, respectively. Of the 17 patients in the R/R group, 11 had a complete response, two had a partial response, one had stable disease, and three had progressive disease or relapsed at the end of treatment. The median OS was 33.1 months and the three-year PFS and OS were 0.75 and 0.86, respectively. Treatment was generally well tolerated, including in patients with co-morbidities and those who had experienced toxicities with previous therapies. These findings demonstrate that the use of dinutuximab beta is feasible and beneficial as a first-line or R/R treatment in routine clinical practice in Poland.

## 1. Introduction

Anti-GD2 monoclonal antibodies are the standard of care for maintenance treatment in the first-line setting for patients with high-risk neuroblastoma (HR-NB) [1]. The first randomized trial demonstrating improved survival with anti-GD2 antibodies compared with standard therapy in patients with HR-NB was the ANBL0032 study of the Children’s Oncology Group (COG) [2]. Maintenance therapy with dinutuximab, a human/mouse chimeric anti-GD2 antibody (ch14.18), plus alternating cycles of granulocyte–macrophage colony-stimulating factor (GM-CSF) and interleukin-2 (IL-2), significantly improved two-year event-free survival (EFS; 66% versus 46%, *p* = 0.01) and overall survival (OS; 86% versus 75%, *p* = 0.02) [2]. Later, in clinical trials conducted by the International Society of Paediatric Oncology European Neuroblastoma (SIOPEN) Group, dinutuximab beta, an anti-GD2 antibody produced in Chinese hamster ovary cells (ch14.18/CHO), plus isotretinoin with or without IL-2, resulted in a similar survival benefit in patients with HR-NB compared with historical controls [1,3,4]. The same studies also showed that the addition of IL-2 to dinutuximab beta plus isotretinoin increased toxicity but did not improve outcomes, and as a result, SIOPEN no longer recommends the use of IL-2 in the first-line setting [1,3,4].

Currently, there is no standard therapy for patients with relapsed or refractory (R/R) neuroblastoma, who are known to have a poor prognosis [5]. Indeed, many patients do not respond to treatment for R/R disease, which generally includes chemotherapy combinations that have not been used previously, with or without consolidation myeloablative therapy with autologous or haploidentical stem cell transplantation or ^131^I meta-iodobenzylguanidine (MIBG) therapy [5,6,7,8]. Some patients with R/R disease demonstrate an initial treatment response, which is then followed by disease progression [5]. Dinutuximab beta maintenance therapy (with isotretinoin ± IL-2) demonstrated benefits in patients with stable R/R HR-NB in the long-term infusion (LTI) SIOPEN trial, and the addition of IL-2 was once again shown to be of no additional benefit [9]. Furthermore, maintenance therapy with single-agent dinutuximab beta was recently shown to be effective in the management of patients with R/R HR-NB, with a clinically meaningful response rate [10].

Dinutuximab beta was approved in Europe in May 2017 for the treatment of HR-NB in patients ≥12 months of age who have achieved at least a partial response (PR) to induction chemotherapy, followed by myeloablative therapy supported with autologous stem cell transplantation (ASCT), and in patients with R/R neuroblastoma with or without residual disease after stabilization of the disease using other treatment methods [11]. The prescribing information for dinutuximab beta also recommends IL-2 co-administration in patients with R/R HR-NB [11], but it was shown more recently that the addition of IL-2 does not improve outcomes in these patients [4,9]. 

The safety and tolerability of dinutuximab beta maintenance therapy have been improved by its administration via continuous long-term infusion and in the absence of IL-2 [4,9], as well as appropriate prophylaxis before and during administration [11]. However, dinutuximab beta is still commonly associated with adverse events (AEs), such as neuropathic pain, capillary leak syndrome (CLS), and allergic reactions, which require adequate supportive treatment and careful monitoring [12].

Currently, there are limited published data regarding the use of dinutuximab beta in real-world clinical practice [13,14,15]. Real-world data complement those from clinical trials providing further evidence of therapeutic effectiveness and safety in routine clinical practice.

We present data from patients with HR-NB treated with dinutuximab beta maintenance therapy in first-line and the R/R settings in everyday clinical practice in Poland.

## 2. Materials and Methods

A retrospective review of the medical records of patients with HR-NB who received at least one cycle of dinutuximab beta immunotherapy combined with isotretinoin (±subcutaneous IL-2) as standard maintenance therapy in a first-line or the R/R setting in Poland between May 2017 and June 2022 was undertaken. Data were collected from three centers that are members of the Polish Pediatric Solid Tumor Study Group (PPSTSG), which are the only centers that provide dinutuximab beta immunotherapy to patients with HR-NB in Poland: the Department of Pediatric Oncology and Hematology at the University Children’s Hospital of Krakow (Krakow, Poland), the Department of Pediatric Bone Marrow Transplantation, Oncology and Hematology at the Wroclaw Medical University (Wroclaw, Poland), and the Department of Oncology at the Children’s Memorial Health Institute (Warsaw, Poland).

### 2.1. Patients and Treatment

Patients were categorized as having HR-NB based on the International Neuroblastoma Staging System (INSS), i.e., if they were ≥12 months of age and had INSS stage 4 neuroblastoma or if they had INSS stage 2, 3, 4, or 4S neuroblastoma with MYCN amplification at diagnosis, irrespective of age [16,17]. All patients were later re-evaluated and classified according to the International Neuroblastoma Risk Group (INRG) Staging System [18]. Patients were included if they received dinutuximab beta as maintenance treatment in the first-line or the R/R setting. 

First-line patients were treated according to the HR-NBL-1/SIOPEN protocol, consisting of induction therapy followed by consolidation and maintenance therapy, with appropriate local treatment [19]. Induction therapy comprised Rapid COJEC (cisplatin (C), vincristine (O), carboplatin (J), etoposide (E), and cyclophosphamide (C)), with a maximum of two cycles of topotecan–vincristine–doxorubicin (TVD) in the event of an insufficient treatment response [19,20]. Some patients received a maximum of two cycles of other neuroblastoma-related chemotherapy regimens than TVD based on the decision of the treating clinician, and continued treatment according to the protocol once they achieved a sufficient response. Consolidation therapy (myeloablative therapy) comprised busulfan and melphalan (BuMel)—the standard of care in Europe since 2017 [21], followed by ASCT. Maintenance therapy comprised dinutuximab beta immunotherapy with isotretinoin (±IL-2).

HR-NB was considered refractory if patients had received more chemotherapy cycles than permitted (i.e., >two cycles of TVD or other neuroblastoma-related chemotherapy regimens according to the clinician’s decision) and/or additional consolidation treatment, e.g., MIBG therapy due to an insufficient response to induction. HR-NB was considered relapsed in patients whose disease had progressed (new lesions or the progression of existing lesions in patients without complete response (CR)) or relapsed (new lesions following CR) during or after first-line therapy and who received dinutuximab beta maintenance therapy in the second line or later. The treatment of patients with R/R HR-NB before the maintenance phase was not standardized.

In both settings, first-line and R/R, dinutuximab beta was given as a continuous infusion of 10 mg/m^2^/day on days 1–10 of each 35-day cycle, for up to five cycles, as recommended [4,11]. All patients received the recommended supportive treatment during the administration of dinutuximab beta [11]. Isotretinoin at 160 mg/m^2^/day was administered for 14 days for up to six cycles; the first cycle was administered before the first cycle of dinutuximab beta and on days 11–24 of each remaining cycle. In accordance with the prescribing information, IL-2 was only given in the R/R setting [11] and was administered at 3 × 10^6^ IU/m^2^/day for five consecutive days in the week preceding the first cycle of dinutuximab beta and on days 2–6 of each remaining cycle (before the results of the LTI SIOPEN study were published, demonstrating increased toxicity without additional benefit in patients treated with IL-2) [9].

Data from patients who received dinutuximab beta as part of the SIOPEN clinical studies before it was approved in Europe, or as part of the EUSA PASS study, were not included in this analysis.

### 2.2. Assessments and Outcomes

Disease was evaluated routinely prior to initiating immunotherapy, and response was assessed after cycle 2, after the end of treatment, and at any time when progression/relapse was suspected, using the International Neuroblastoma Response Criteria [22]. Tumors were assessed radiographically in patients with measurable disease using computed tomography and/or magnetic resonance imaging. MIBG response was evaluated in patients with iodine-123 or iodine-131-labeled MIBG-positive lesions (before initiating dinutuximab beta, after cycle 2 for patients receiving immunotherapy in the R/R setting, and after cycle 5). Patients with MIBG non-avid disease underwent positron emission tomography. Bone marrow involvement was assessed bilaterally using routine cytomorphologic examination before therapy and after cycles 2 and 5, and histopathologic examination with immunostaining before and after treatment and after cycle 2 in the R/R setting.

Progression-free survival (PFS) was defined as the time from dinutuximab beta initiation to the first occurrence of relapse or disease progression. OS was defined as the time from dinutuximab beta initiation until death with any cause or the date of the last observation.

Patients were monitored for AEs, which were graded according to the Common Terminology Criteria for Adverse Events (CTCAE) version 4.0. Pain was evaluated using the Wong–Baker FACES Pain Rating Scale and the Face, Legs, Activity, Cry, Consolability (FLACC) scale in patients under three years of age, with both scales ranging from 0 (no pain) to 10 (worst pain imaginable) [23,24].

### 2.3. Statistical Analysis

The data cut-off was 31 July 2022. The one-year and three-year PFS and OS probabilities were estimated using the Kaplan–Meier method (patients were censored on the date of the last assessment) and differences between the analyzed groups were compared using a log-rank test (*p* < 0.05 was statistically significant) [25,26]. The effects of the following factors on PFS and OS were analyzed: age <12 months at diagnosis, MYCN amplification, INSS stage of disease (3 versus 4), and the presence of active disease at dinutuximab beta initiation (CR versus non-CR). 

The effects of the following factors on occurrence of death and relapse/progression after dinutuximab beta immunotherapy were analyzed using univariate analysis with logistic regression (odds ratio (OR); *p* < 0.05 was considered statistically significant): age <12 months at diagnosis, MYCN amplification, presence of active disease at dinutuximab beta initiation (CR versus non-CR) and number of metastatic compartments in INSS stage 4 patients at diagnosis (1 versus >1).

The effectiveness of dinutuximab beta was analyzed separately in patients receiving it in the first-line setting and those receiving it in the R/R setting. For the R/R setting, risk factor analyses were performed for the whole group, but PFS and OS were also analyzed separately for refractory and relapsed patients. Safety data were analyzed for patients treated in the first-line and the R/R settings combined.

## 3. Results

### 3.1. Dinutuximab Beta in the First-Line Setting

#### 3.1.1. Patient Characteristics

In total, 37 patients with HR-NB who received dinutuximab beta maintenance therapy in the first-line setting were studied. No patients were lost to follow-up. The patient demographics and disease characteristics are shown in Table 1. Most patients (*n* = 24, 64.9%) were male and the median age was 34.77 months (interquartile range (IQR) 19.2–50.6). MYCN was amplified in 18 (48.6%) patients, including four patients <12 months of age. The majority of patients (*n* = 33, 89.2%) had INSS stage 4 (M) neuroblastoma at diagnosis, and four (10.8%) had INSS stage 3 (L2) disease with MYCN amplification.

The primary tumor was most often localized in the abdomen (86.5%). At diagnosis, 26 (70.3%) patients had metastases in two or more compartments, with the most common sites being the bone marrow (73.0%) and bones (70.3%). In patients with metastases in a single compartment, the bones (*n* = 3), bone marrow (*n* = 3), and lymph nodes (*n* = 1) were affected.

All patients were treated according to the HR-NBL-1/SIOPEN protocol. Chemotherapy for nephroblastoma or soft tissue tumors was administered before COJEC in two patients, due to diagnostic difficulties. Additional chemotherapy due to an insufficient response to COJEC was administered in 21 (56.8%) patients, most commonly TVD (*n* = 16, 43.2%), with three patients receiving TEMIRI (irinotecan/temozolomide), one receiving PACE (teniposide, doxorubicin, cyclophosphamide, cisplatin), and one receiving etoposide/carboplatin. After additional chemotherapy (maximum two cycles), the patients demonstrated sufficient responses and continued therapy according to the HR-NBL-1/SIOPEN protocol. CR was achieved in 30 (81.1%) patients and PR in seven (18.9%) prior to initiating dinutuximab beta maintenance therapy.

All five planned cycles of dinutuximab beta were received by 29 (78.4%) patients. Of the eight patients who received fewer than five cycles of dinutuximab beta, one died due to sepsis complications and seven had progressive disease (PD) or relapsed. An additional cycle of dinutuximab beta was received by one patient who had received a drastically reduced dose during cycle 3 due to an anaphylactic reaction.

#### 3.1.2. Outcomes

In total, 12 (32.4%) patients relapsed, with a median time to relapse of 20.07 months (IQR 9.8–36.6). Seven patients relapsed during first-line dinutuximab beta immunotherapy (18.9% of all patients, 58.3% of all relapsed patients) and five after the end of treatment. All relapses were metastatic, including four patients (33.3%) with relapses in the central nervous system (CNS), three of whom had an isolated CNS relapse during immunotherapy, and one who had a parenchymal brain lesion and bone lesions three months after immunotherapy. All patients with CNS involvement progressed and eventually died of disease. Of the seven patients who relapsed during dinutuximab beta immunotherapy, five died due to first PD and two achieved a second CR with consecutive relapse. One of them died due to PD and the other is still being treated for their second relapse as of March 2023. Of the five patients who relapsed following dinutuximab beta therapy, two died due to PD and three received immunochemotherapy (dinutuximab beta plus temozolomide and irinotecan), one of whom achieved a CR and two a PR. All patients who relapsed after immunotherapy had a CR at the end of dinutuximab beta treatment.

Of the 30 patients who had a CR prior to initiating dinutuximab beta immunotherapy, 26 still had a CR at the end of therapy and four experienced a relapse. Of the seven patients with a PR before immunotherapy, two patients achieved CR (28.6%), two had SD and thus remained in PR, and three had PD at the end of treatment.

In total, nine patients (24.3%) died, one due to septic complications (2.7%) and eight (21.6%) due to PD. The median survival was 24.37 months (IQR 9.9–37.8). The PFS ± standard error (SE) was 0.75 ± 0.07 at one year and 0.63 ± 0.09 at three years, and the OS ± SE at one year and three years were 0.83 ± 0.06 and 0.80 ± 0.15, respectively. 

**Figure 1:**
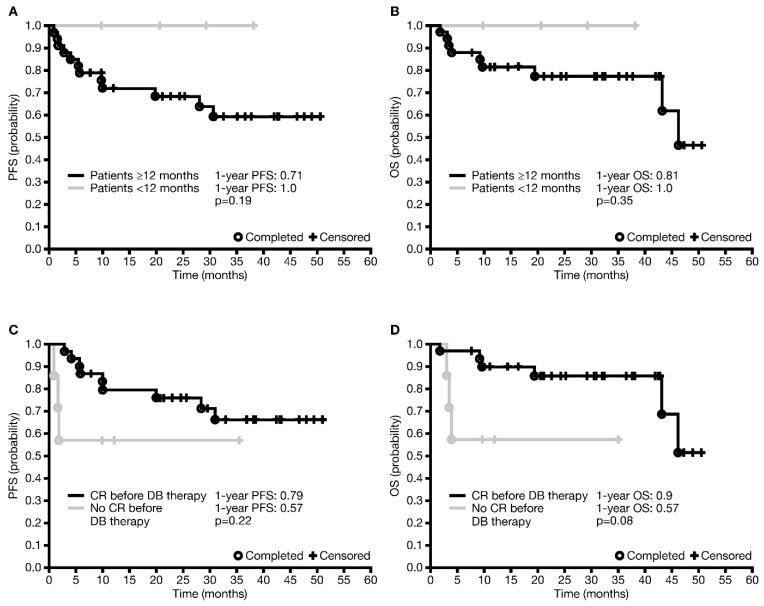
Factors affecting survival in patients who received dinutuximab beta maintenance therapy in the first-line setting. (**A**) PFS by age <12 months or ≥12 months at diagnosis. (**B**) OS by age <12 months or ≥12 months at diagnosis. (**C**) PFS in the absence or presence of active disease prior to initiating immunotherapy. (**D**) OS in the absence or presence of active disease prior to initiating immunotherapy. CR, complete response; DB, dinutuximab beta; OS, overall survival; PFS, progression-free survival.

In the analysis of factors affecting survival, neither one-year PFS nor OS were statistically significantly affected by MYCN amplification (*p* = 0.44 and *p* = 0.32, respectively), or disease stage (INSS stage 3 versus 4: *p* = 0.43 and *p* = 0.11, respectively). There were also no statistically significant differences in one-year PFS or OS based on age at diagnosis (<12 versus ≥12 months; *p* = 0.19 and *p* = 0.35, respectively; Figure 1A,B); however, all patients <12 months of age at diagnosis were alive and disease-free one and three years after initiating dinutuximab beta. The one-year PFS was also not significantly affected by the presence of active disease (less than CR) when initiating immunotherapy (*p* = 0.22; Figure 1C). While it appeared that more patients who achieved a CR prior to initiating immunotherapy were alive at one year than those who did not, the difference was also not statistically significant (*p* = 0.08; Figure 1D). None of the factors investigated had any statistically significant influence on the occurrence of disease relapse/progression or death (Table S1).

### 3.2. Dinutuximab Beta in the Relapsed/Refractory Setting

#### 3.2.1. Patient Characteristics

In total, 17 patients with HR-NB who received dinutuximab beta maintenance therapy in the R/R setting were studied, including eight with relapsed disease and nine with refractory disease. No patients were lost to follow-up. The patient demographics and disease characteristics are shown in Table 1. The majority of patients (*n* = 13, 76.5%) were male, the median age was 39.83 months (IQR 25.3–58.0), and MYCN was amplified in three (17.6%) patients. Most patients (*n* = 13, 76.5%) had INSS stage 4 neuroblastoma at diagnosis, including three patients <12 months of age, two of whom had MYCN amplification; the one without MYCN amplification was considered HR due to stage 4 refractory disease with progression in first-line therapy. Four patients were INSS stage 3 at diagnosis and were treated with the HR protocol due to disseminated (*n* = 3) or consecutive local relapses (*n* = 1). The primary tumor was most often localized in the abdomen (88.2%) and metastases were present in two or more compartments in 64.7% of patients, most often in the bone (82.4%), bone marrow (64.7%), and lymph nodes (47.1%). In patients in whom metastasis affected only a single compartment, the bone (*n* = 2), soft tissue (*n* = 1), and lymph nodes (*n* = 2) were affected. 

Of the 17 patients with R/R disease, 12 had received first-line induction and consolidation therapy according to the HR-NBL-1/SIOPEN protocol. Only one of these patients had been treated with first-line dinutuximab beta plus IL-2 as a part of a clinical trial. Five patients received initial chemotherapy according to the LINES protocol [27]. 

The treatment of patients with R/R HR-NB was not standardized, but all patients received chemotherapy (4–21 cycles; Table 2). ^131^I-MIBG therapy with ASCT was given in seven (41.2%) patients (relapsed: *n* = 4; refractory: *n* = 3). Myeloablative therapy with ASCT for the treatment of R/R disease was received by all patients with refractory disease (BuMel according to the HR-NBL-1/SIOPEN protocol) and four patients with relapsed disease who did not have BuMel in the first-line setting. All nine patients with refractory disease and one patient with relapsed disease received radiotherapy. Surgery was undertaken in all refractory and two relapsed patients. A CR was achieved in eight (47.1%) patients prior to initiating dinutuximab beta maintenance therapy.

Dinutuximab beta was given with isotretinoin in all patients; one of them also received IL-2 before the results from the LTI SIOPEN study were published, demonstrating increased toxicity without additional survival benefit in patients treated with IL-2 [9]. GD2 expression was not evaluated prior to dinutuximab beta treatment due to a lack of validated methods analyzing GD2 expression on tumor tissue in the treatment centers.

All five planned cycles of dinutuximab beta were received by 16 (94.1%) patients; one patient stopped therapy after two cycles due to PD. In addition, one patient who received a drastically reduced dose during cycle 1 due to grade 3 CLS received an additional cycle of dinutuximab beta.

#### 3.2.2. Outcomes

At the end of dinutuximab beta maintenance therapy in the R/R setting, 11 (64.7%) patients had a CR, including five out of nine patients (55.6%; 29.4% of all R/R patients) who had active disease before receiving immunotherapy. A further two (11.8%) patients had a PR, resulting in an objective response rate of 76.5%. One (5.9%) patient remained in SD and three (17.6%) patients experienced relapse or PD at the end of immunotherapy, two of whom had a CR and one of whom had active disease prior to immunotherapy. In addition, one of the patients who achieved CR relapsed 19.8 months after initiating immunotherapy. All four relapses were metastatic, with a median time to relapse of 6.10 months (IQR 12.8–46.2). Two of the four patients who relapsed died due to PD. 

**Figure 2:**
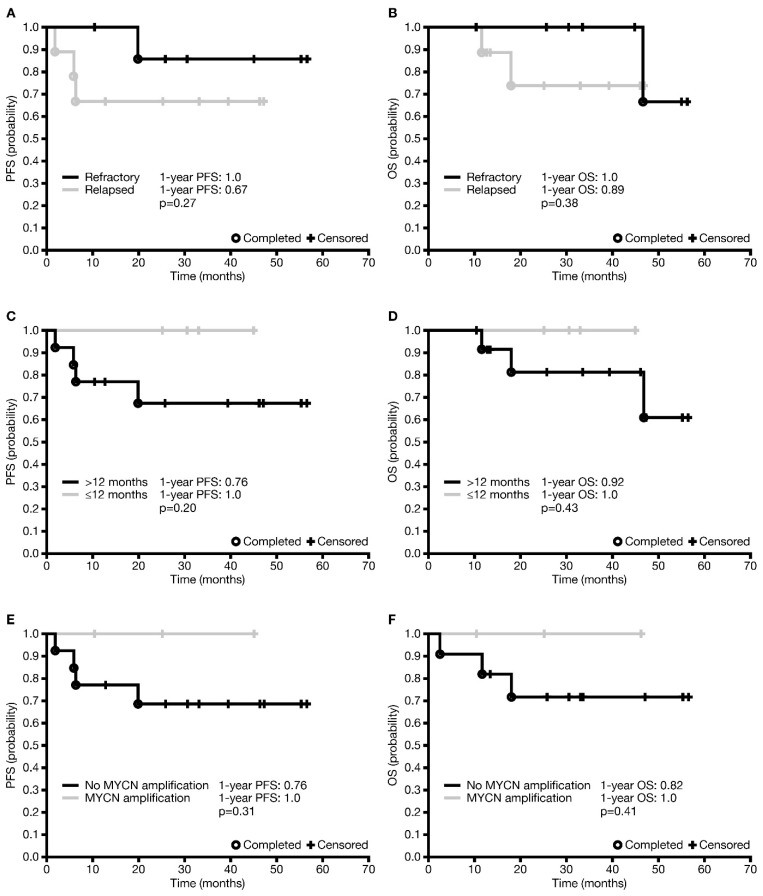
Factors affecting survival in patients who received dinutuximab beta maintenance therapy in the relapsed/refractory setting. (**A**) PFS in patients with refractory disease versus relapsed disease. (**B**) OS in patients with refractory disease versus relapsed disease. (**C**) PFS by age <12 months or ≥12 months at diagnosis. (**D**) OS by age <12 months or ≥12 months at diagnosis. (**E**) PFS in patients with and without MYCN amplification. (**F**) OS in patients with and without MYCN amplification. OS, overall survival; PFS, progression-free survival.

In total, three (17.6%) patients died—two from PD and one from late complications (lung fibrosis) most probably unrelated to dinutuximab beta, 47 months after starting immunotherapy. The median survival was 33.1 months (IQR 18.0–46.2). The one-year and three-year PFS ± SE were 0.82 ± 0.09 and 0.75 ± 0.10, respectively. The difference in one-year PFS between patients with relapsed disease and those with refractory disease was not statistically significant (*p* = 0.27), although it appeared that more patients with refractory disease were progression-free than those with relapsed disease (Figure 2A). The one-year OS ± SE was 0.94 ± 0.06 and the three-year OS was 0.86 ± 0.10 for the entire R/R cohort. However, the difference in one-year OS between refractory and relapsed patients was not statistically significant (*p* = 0.38; Figure 2B).

An analysis of factors affecting survival was undertaken in all patients who received dinutuximab beta in the R/R setting, and not separately in those with relapsed disease and those with refractory disease, due to the relatively low number of patients and events in the individual groups. There was no statistically significant difference in one-year PFS or OS between patients based on age at diagnosis (<12 versus ≥12 months; *p* = 0.20 and *p* = 0.43 (Figure 2C,D), respectively), but no events were observed in patients <12 months at diagnosis. One-year PFS and OS were statistically significantly different neither in patients with or without MYCN amplification (Figure 2E,F; *p* = 0.31 and *p* = 0.41, respectively) nor in patients with CR or non-CR prior to immunotherapy (*p* = 0.78 and *p* = 0.87, respectively). Analysis depending on INSS stage was not carried out due to a very low number of patients in stage 3. None of the factors investigated had any statistically significant influence on the occurrence of disease relapse/progression or death (Table S2).

### 3.3. Safety

Dinutuximab beta was well tolerated, with relatively few clinically relevant AEs (Table 3; Table S3). The occurrence of AEs was similar in the first-line and R/R settings, and between patients with CR and non-CR prior to immunotherapy. While in most patients, premedication with gabapentin and continuous infusion of morphine and metamizole successfully prevented pain, it was reported in 15 of 54 (27.8%) patients during cycle 1. Of these, seven (46.7%) patients had pain rated as 1–3 on a 10-point scale, four (26.7%) had pain rated as 4–6, and four (26.7%) had pain rated as 7–8. Pain was less severe and occurred in fewer patients in consecutive cycles (Table S3), and generally responded to standard treatment provided at the respective centers (additional paracetamol doses, a morphine bolus or an increased basal morphine infusion rate). Dinutuximab beta was not stopped due to pain in any patient. One patient had pain rated as 10/10 in cycle 2, which was caused by PD and compression of the spinal cord and was not associated with dinutuximab beta.

CLS was reported in 42 (77.89%) patients during cycle 1 and was predominantly graded as 1 or 2 (38/42; Table 3). Grade 3 CLS (but without severe hypotension) was reported in four patients, resulting in the temporary discontinuation of dinutuximab beta treatment. Dinutuximab beta was well tolerated in subsequent cycles in these patients, although the dosage in consecutive cycles was slowly increased until the full dose was reached.

Only one severe allergic reaction (anaphylaxis with hypotension) was observed, which resulted in the patient temporarily stopping therapy during cycle 3 after receiving 15% of the dose. The patient also had mild allergic reactions during cycles 1 and 2, responding to supportive therapy. Therapy was continued up to six cycles of dinutuximab beta without additional toxicities. Grade 3 allergic toxicity (bronchial constriction) was reported in three other patients and was effectively managed with supportive treatment. Other common allergic reactions included pruritus, sneezing with nasal discharge, and skin exanthema.

No severe neurologic AEs were reported. Mydriasis grade 1 was observed in 20 (37.0%) patients; four (7.4%) had visual impairment that did not worsen during consecutive cycles and required correction with glasses. One patient with adrenal insufficiency and ion disturbances had convulsions during cycles 3 and 4. Problems with miction were reported in four patients during cycle 1, and resolved after morphine discontinuation/naloxone administration, but they were considered unrelated to dinutuximab beta. Due to metamizole premedication, only three patients had grade 3 fever, all during cycle 1. Only a few infections occurred and were mostly manageable. There were two cases of sepsis; one patient had sepsis after cycle 1, which was treated successfully, and the other had sepsis due to *Escherichia coli* during cycle 2 and died of complications (bleeding into the CNS). Two patients had AEs that required dose reductions (90% of the dinutuximab beta dose); one patient experienced severe vomiting and diarrhea during cycle 1 that required parenteral nutrition, and one had hypertension during cycle 3. 

Two patients had diarrhea during each cycle of therapy—one had increased vasoactive intestinal peptide excretion and the other had gluten enteropathy, both diagnosed during treatment. Other co-morbidities included an atrial septal defect, chronic kidney disease (grade 2), adrenal insufficiency in two patients, and a Factor VII deficiency, which did not influence the course of immunotherapy. Dinutuximab beta therapy was also feasible in heavily pretreated patients with severe complications of previous therapy (chronic ischemic pancreatitis and hepatitis, previous cardiac arrest, gastrointestinal tract obstruction, and acute kidney failure). 

Hematologic and biochemical abnormalities due to dinutuximab beta therapy were also observed and were, in most cases, managed by correcting ion levels or by administering erythrocytes or platelet concentrates. Low albumin levels were not routinely corrected. Two patients required temporary discontinuation of dinutuximab beta treatment due to grade 4 increases in transaminases. C-reactive protein levels were increased in most patients during cycle 1 (median 136 mg/L; normal: <10 mg/L, with a tendency to normalize in consecutive cycles). Patients with neutropenia and fever were monitored closely; routine antibiotics were not given. 

The most commonly reported late AEs (i.e., AEs that occurred after therapy completion) were thyroid insufficiency and ototoxicity. We also observed one case of lung fibrosis leading to death almost four years after the beginning of immunotherapy, but it was probably unrelated to dinutuximab beta treatment.

## 4. Discussion

The results of this retrospective chart review demonstrate the feasibility and clinical benefits of dinutuximab beta maintenance therapy in 54 patients with HR-NB, including 37 who received it in a first-line setting and 17 who received it in an R/R setting, as part of routine clinical practice at one of three pediatric oncology centers in Poland.

The efficacy of anti-GD2 antibodies has been demonstrated in clinical trials, leading to their incorporation into the standard of care maintenance treatment of patients with HR-NB [1,2,3,28]. In our analysis of real-world data, patients who received dinutuximab beta maintenance therapy with isotretinoin in the first-line setting had a three-year PFS ± SE of 0.63 ± 0.09 and a three-year OS ± SE of 0.80 ± 0.15, which are comparable to the results achieved in the SIOPEN study [1]. In that study, immunotherapy with dinutuximab beta resulted in a five-year EFS rate of 57% (95% CI 51–62%) and a five-year OS rate of 64% (95% CI 59–69%) in 378 patients with HR-NB, which was significantly better than the rates reported for a historical control group treated without immunotherapy (*p* < 0.001) [1]. The most important factors significantly influencing EFS in patients of the SIOPEN study treated with immunotherapy were INSS stage and the number of metastatic compartments involved [1]. In our cohort, we analyzed the probability of survival and the risk of relapse/progression and death based on age at diagnosis, MYCN amplification, INSS stage, and active disease prior to immunotherapy. However, none of these factors demonstrated a statistically significant influence on treatment results, which may be due to the relatively low number of patients included in our study. Interestingly, we did not observe any events in children younger than 12 months at diagnosis. The worst prognosis was seen in patients who relapsed during or just after finishing therapy with dinutuximab beta. As three out of seven relapsed patients (42.9%) had isolated CNS disease, such clinical events must be taken into consideration in children with sudden onset of neurological symptoms. Although an increased incidence of CNS relapse was not confirmed in the SIOPEN cohort treated with immunotherapy [29], all patients with neurological symptoms must be strictly monitored to confirm its cause, especially in children under the age of 12 months with MYCN amplification, who appear to have an increased risk for early CNS relapse [30].

The efficacy of dinutuximab beta therapy has also been demonstrated in R/R patients in whom disease was stabilized using other methods—both in combination with isotretinoin [9] and as monotherapy [10]. In the study investigating dinutuximab beta monotherapy, the objective response rate was 53% including minor responses, and the three-year PFS and three-year OS rates were 31.5% and 65.5%, respectively [10]. Combination therapy with dinutuximab beta and isotretinoin resulted in a two-year EFS rate of 59% and a two-year OS rate of 79%, which were not significantly improved when IL-2 was added to the regimen [9]. In our analysis, immunotherapy with dinutuximab beta and isotretinoin in the R/R setting was associated with a three-year PFS ± SE of 0.75 ± 0.1 and a three-year OS of 0.86 ± 0.1. While the difference in PFS between patients with relapsed disease and those with refractory disease was not statistically significant (*p* = 0.27), probably due to a low number of patients, more patients with refractory disease appeared to be progression-free than those with relapsed disease. Other studies demonstrated better survival in patients with refractory disease than in those with relapsed disease when dinutuximab beta was used as a maintenance treatment [9].

Maintenance treatment with dinutuximab beta has been shown to result in further disease regression in patients with PR prior to immunotherapy [9]. In our study, dinutuximab beta treatment in the first-line setting resulted in CR in two out of seven (28.6%) patients with PR prior to immunotherapy. Similarly, in the R/R setting, five out of nine patients (55.6%) with PR before immunotherapy achieved CR. The LTI SIOPEN study reported CRs of 26% and 9% in R/R patients with active disease prior to immunotherapy who were treated with DB and isotretinoin with or without IL-2, respectively [9]. These findings show that immunotherapy is not only effective in the treatment of minimal residual disease, but also measurable disease. In all of the analyses discussed here, patients with metastatic and primary lesions were included.

As recommended by SIOPEN [4] and the prescribing information for dinutuximab beta [11], the majority of patients received five cycles of dinutuximab beta. However, it is not clear whether additional cycles might be helpful if there is still residual disease after the end of planned therapy. Although late responses are observed after the end of dinutuximab beta therapy, it is difficult to predict in which patients they may occur, and in whom the progression of disease may develop. Taking into consideration the very low toxicity of the consecutive cycles of dinutuximab beta, additional cycles might be considered in patients who are responding but do not have a CR at the end of the planned treatment. In our cohort, an additional cycle of dinutuximab beta was administered in two patients who received lower doses during an earlier cycle because of severe adverse events (anaphylaxis and CLS) without the occurrence of further toxicities.

Dinutuximab beta was generally well tolerated, with AEs similar to those observed in clinical trials [1,3,4]. Observed grade 3 toxicities were manageable and, in most cases, did not require therapy to be discontinued. In line with data from previous studies [3,10,31], the tolerability profile of dinutuximab beta generally improved with subsequent cycles, and most AEs were manageable with standard supportive therapy. Pain, which is one of the most common AEs associated with anti-GD2 antibodies [12,32], was well managed with analgesics given according to institutional guidelines. The long-term infusion schedule that we used in our patients has previously been shown to result in reduced pain compared with short-term infusion and was generally well tolerated [9,10]. In addition, we observed no severe neurological disturbances—mydriasis and visual disturbances were reversible and did not require discontinuation of therapy. Although rare, severe neurological AEs have been reported to occur in patients treated with dinutuximab beta [33]. Patients should therefore be strictly observed by experienced healthcare professionals who can react immediately to any clinical or laboratory changes to prevent the development of more severe and potentially irreversible conditions. While therapy should be stopped in cases of severe neurotoxicity, we suggest considering a rechallenge with dinutuximab beta in patients with other severe toxicities, due to the poor prognosis of patients with HR-NB who do not receive immunotherapy [1,2]. If attempted, appropriate prophylaxis should be administered, and the patient should be monitored closely. Indeed, therapy was successfully continued in two of our patients who experienced a severe allergic reaction or CLS. 

Although the toxicity profile of dinutuximab beta is well described, there are no clear guidelines for its inclusion anduse in the treatment of patients with co-morbidities or those who have had severe toxicities during previous therapy. We demonstrated in our study that these patients can also be safely treated with dinutuximab beta, with careful monitoring and adjustment of supportive therapy.

To our knowledge, we report data from the largest cohort of real-world patients treated with dinutuximab beta maintenance therapy to date, with relatively long follow-up (up to five years). However, due to the retrospective design of this study and the lack of a comparison group, the value of the conclusions drawn from this study may be limited. 

In conclusion, the results of this study complement the evidence from clinical trials and confirm the real-world benefits of maintenance therapy with dinutuximab beta in patients with HR-NB, both in the first-line and R/R settings. These results also confirm the feasibility of including dinutuximab beta in the multimodal treatment of these patients in routine clinical practice, including patients with co-morbidities and those who experienced toxicities with previous therapy. While most AEs are predictable and manageable with standard supportive therapy, severe AEs may occur that require immediate action, suggesting that dinutuximab beta should be provided at centers experienced in its administration.

## Figures and Tables

**Table 1:** Patient and disease characteristics.

	Dinutuximab Beta in the First-Line Setting *n* (%)	Dinutuximab Beta in the R/R Setting *n* (%)
Total number of patients	37	17
Median age at diagnosis (IQR), months	34.77 (19.2–50.6)	39.83 (25.3–58.0)
Patients <12 months of age	4 (10.8)	4 (23.5)
Male	24 (64.9)	13 (76.5)
MYCN amplification		
Yes	18 (48.6)	3 (17.6)
No	15 (40.5)	13 (76.5)
Unknown	4 (10.8)	1 (5.9)
INSS stage at diagnosis		
Stage 3	4 (10.8)	4 (23.5) ^a^
Stage 4	33 (89.2)	13 (76.5)
INRG stage at diagnosis		
Stage L3	4 (10.8)	4 (23.5) ^a^
Stage M	33 (89.2)	13 (76.5)
Primary tumor localization		
Abdomen	32 (86.5)	15 (88.2)
Chest	3 (8.1)	2 (11.8)
Neck	2 (5.4)	0 (0)
Metastases ^b^		
Bone marrow	27 (73.0)	11 (64.7)
Bone	26 (70.3)	14 (82.4)
Lymph nodes	14 (37.8)	8 (47.1)
Other ^b^	6 (16.2) ^c^	3 (17.6) ^d^
Number of metastatic compartments		
0	4 (10.8)	1 (5.9)
1	7 (18.9)	5 (29.4)
2	13 (35.1)	4 (23.5)
3	11 (29.7)	7 (41.2)
4	2 (5.4)	0 (0)
Status before initiating dinutuximab beta		
CR	30 (81.1)	8 (47.1)
PR	7 (18.9)	9 (52.9)

CR, complete response; INRG, International Neuroblastoma Risk Group; INSS; International Neuroblastoma Staging System; IQR, interquartile range; PR, partial response; R/R, relapsed/refractory. ^a^ Two patients >18 months of age who had stage 3 (L2) disease without MYCN amplification at diagnosis received dinutuximab beta maintenance therapy for metastatic disease. ^b^ Patients could have metastases at more than one site/compartment. ^c^ Liver (*n* = 4); lungs (*n* = 2), central nervous system (*n* = 1), meninges (dura; *n* = 1), kidney (*n* = 1), skin (*n* = 1). ^d^ Liver (*n* = 2), central nervous system (*n* = 1).

**Table 2:** Treatment received by patients for relapsed/refractory high-risk neuroblastoma.

	Relapsed *n* (%)	Refractory *n* (%)
Total number of patients	8	9
TVD	3 (37.5)	8 (88.9)
PACE	1 (12.5)	4 (44.4)
TEMIRI	5 (62.5)	4 (44.4)
TOTEM	2 (25.0)	1 (11.1)
Rapid COJEC (second-line)	2 (25.0)	1 (11.1)
High-dose cyclophosphamide	1 (12.5)	1 (11.1)
Chemotherapy plus bevacizumab	3 (37.5)	0 (0)
DIP	2 (25.0)	0 (0)
Etoposide/dacarbazine	1 (12.5)	0 (0)
Topotecan/cyclophosphamide	1 (12.5)	0 (0)
BuMel with ASCT	4 (50.0)	9 (100.0)
^131^I MIBG therapy with ASCT	4 (50.0)	3 (33.3)

^131^I MIBG, iodine-131-labeled meta-iodobenzylguanidine; ASCT, autologous stem cell transplant; BuMel, busulfan and melphalan COJEC, cisplatin, vincristine, carboplatin, etoposide, and cyclophosphamide; DIP, dacarbazine, etoposide + vincristine, cyclophosphamide + PACE; PACE, teniposide, doxorubicin, cyclophosphamide, and cisplatin; TEMIRI, irinotecan, temozolomide; TOTEM, topotecan, temozolomide; TVD, topotecan, vincristine, doxorubicin.

**Table 3:** Adverse events in patients who received dinutuximab beta first-line or in the relapsed/refractory setting (*n* = 54).

Adverse Event	Grade	Dinutuximab Beta Cycle				
		1	2	3	4	5
Anemia	1	4	5	3	6	8
	2	18	20	16	12	11
	3	22	12	11	8	3
	4	1	1	2	0	0
Thrombocytopenia	1	6	3	1	3	1
	2	7	3	2	3	1
	3	9	5	5	2	0
	4	3	4	1	1	0
Neutropenia	1	8	5	2	9	2
	2	8	8	9	6	6
	3	8	8	11	6	3
	4	4	6	1	0	1
Increased transaminases (ALT and/or AST)	1	13	16	12	15	7
	2	8	12	8	5	3
	3	13	7	5	3	9
	4	1	0	1	1	0
Capillary leak syndrome	1	15	6	4	1	1
	2	23	7	4	1	0
	3	4	0	0	0	0
	4	0	0	0	0	0
Fever	1	3	3	1	1	1
	2	9	5	1	1	3
	3	3	0	0	0	0
	4	0	0	0	0	0
Allergic reaction	1	3	2	2	3	2
	2	8	4	3	3	1
	3	4	1	1	3	0
	4	0	0	1	0	0
Hypertension	1	0	0	0	0	0
	2	0	0	1	0	0
	3	0	0	0	0	0
	4	0	0	0	0	0
Hypotension	1	0	1	0	1	0
	2	0	0	0	0	0
	3	4	0	0	0	0
	4	0	0	0	0	0
Mydriasis	1	8	7	4	1	0
	2	0	0	0	0	0
	3	0	0	0	0	0
	4	0	0	0	0	0
Infection	1	0	3	9	2	4
	2	7	1	0	0	0
	3	3	2	1	3	3
	4	1	1	0	0	0
Diarrhea	1	5	1	2	4	1
	2	14	4	3	2	2
	3	1	0	0	0	0
	4	0	0	0	0	0
Nausea/vomiting	1	2	1	1	1	0
	2	1	0	0	0	0
	3	0	0	0	0	0
	4	1	0	0	0	0

ALT, alanine aminotransferase; AST, aspartate aminotransferase. Data present number of patients with graded adverse events.

## Data Availability

The original contributions presented in this study are included in the article/Supplementary Materials. Further inquiries can be directed to the corresponding author.

## Supplementary Materials

The following supporting information can be downloaded at: https://www.mdpi.com/article/10.3390/jcm12165252/s1, Table S1: Factors affecting the odds of relapse or death in patients who received dinutuximab beta maintenance therapy in the first-line setting; Table S2: Factors affecting the odds of relapse or death in patients who received dinutuximab beta maintenance therapy in the relapsed/refractory setting; Table S3: Pain during dinutuximab beta therapy, rated on a 10-point (0 (no pain) to 10 (worst pain imaginable); *n* = 54).
