# Supplementary material for: Dinutuximab Beta Maintenance Therapy in Patients with High-Risk Neuroblastoma in First-Line and Refractory/Relapsed Settings—Real-World Data
Source: J Clin Med. 2023 Aug 11;12(16):5252. doi: 10.3390/jcm12165252 (PMC10455178; doi:10.3390/jcm12165252)
Supplement: Supplementary file 1 [file jcm-12-05252-s001.zip › jcm-2530271-supplementary.pdf]

Supplementary Materials

Table S1. Factors affecting the odds of relapse or death in patients who received dinutuximab beta maintenance therapy in the first-line setting

	Relapse		Death	
	**OR (95% CI)**	***p*-value**	**OR (95% CI)**	***p*-value**
Age (≥12 versus <12 months at diagnosis)	5.23 (0.26–105.5)	0.28	3.49 (0.17–71.25)	0.42
INSS disease stage (4 versus 3)	0.44 (0.05–3.54)	0.44	0.27 (0.03–2.27)	0.23
MYCN amplification (yes versus no)	1.75 (0.40–7.73)	0.46	3.25 (0.54–19.3)	0.19
Active disease prior to initiating dinutuximab beta (yes versus no)	1.75 (0.32–1.94)	0.52	3.00 (0.52–17.16)	0.21
Number of metastatic compartments involved at diagnosis (>1 versus 1)	0.52 (0.09–2.93)	0.46	0.63 (0.09–4.22)	0.63

CI, confidence interval; INSS, International Neuroblastoma Staging System; OR, odds ratio. Univariate analysis with logistic regression ($p < 0.05$ considered statistically significant).

Table S2. Factors affecting the odds of relapse or death in patients who received dinutuximab beta maintenance therapy in the relapsed/refractory setting

	Relapse		Death	
	**OR (95% CI)**	***p*-value**	**OR (95% CI)**	***p*-value**
Age (≥12 versus <12 months at diagnosis)	4.26 (0.19–97.48)	0.36	4.26 (0.19–97.48)	0.36
INSS disease stage (4 versus 3)	0.25 (0.01–5.26)	0.37	0.15 (0.01–3.58)	0.24
MYCN amplification (yes versus no)	0.30 (0.01–7.17)	0.46	0.66 (0.02–17.18)	0.80
Active disease prior to initiating dinutuximab beta (yes versus no)	0.86 (0.09–8.07)	0.89	2.00 (0.15–27.4)	0.60

CI, confidence interval; INSS, International Neuroblastoma Staging System; OR, odds ratio. Univariate analysis with logistic regression ($p < 0.05$ considered statistically significant).

Table S3. Pain during dinutuximab beta therapy, rated on a 10-point (0 [no pain] to 10 [worst pain imaginable]; *n* = 54)

	**Dinutuximab beta cycle**				
**Pain rating**	**1**	**2**	**3**	**4**	**5**
**1**	0	1	0	1	0
**2**	2	3	1	1	0
**3**	5	2	0	0	0
**4**	1	0	0	0	0
**5**	3	0	0	0	0
**6**	0	0	0	0	0
**7**	2	0	0	0	0
**8**	2	0	0	0	0
**9**	0	0	0	0	0
**10**	0	1 [a]	0	0	0

Data present number of patients. [a] Patient had progressive disease and compression of the spinal cord.
